# The mediating role of environmental restorativeness between vegetation levels and physical activity intention: a photo-based experimental study

**DOI:** 10.3389/fpubh.2025.1653065

**Published:** 2025-10-03

**Authors:** Shici Weng, Xing Zhang, Mingyue Yin, Haodong Tian, Haowei Liu, Hansen Li

**Affiliations:** ^1^Physical Education College, Xichang University, Xichang, China; ^2^Department of Physical Education and Sport, Faculty of Sport Sciences, University of Granada, Granada, Spain; ^3^School of Athletic Performance, Shanghai University of Sport, Shanghai, China; ^4^College of Physical Education, Southwest University, Chongqing, China; ^5^School of Physical Education, Sichuan Agricultural University, Ya’an, China

**Keywords:** restoration, restorativeness, mental health, physical activity, greenspace

## Abstract

**Introduction:**

Vegetation is linked to better health partly by promoting physical activity, but the psychological mechanisms remain unclear. We examined whether perceived environmental restorativeness mediates the association between vegetation level and intention to be physically active.

**Methods:**

In an image-based randomized experiment, Chinese university students viewed simulated outdoor scenes depicting low to high vegetation coverage. After each exposure, participants reported perceived restorativeness (Chinese Perceived Restorativeness Scale; reliability, structural validity, and concurrent validity assessed) and intention to be physically active in the depicted setting. Associations among vegetation level, perceived restorativeness, and intention were tested, and mediation analyses evaluated indirect effects through PRS subscales and the total score.

**Results:**

Greater vegetation coverage was associated with higher perceived restorativeness and stronger intention to engage in physical activity. All PRS subscales significantly mediated the vegetation–intention relationship. The PRS total score showed a full mediation effect, indicating that vegetation influenced physical activity intention largely through perceived restorativeness.

**Discussion:**

Findings identify environmental restorativeness as a key psychological pathway linking vegetation to physical activity motivation and suggest that enhancing restorative qualities may be a practical strategy for green-space design to promote activity. Generalizability is limited by the student sample and simulated scenes; future work should recruit more diverse populations and use ecologically valid environments.

## 1 Introduction

In the era of global urbanization, most people now live in cities (1). This shift has heightened concerns about human–nature disconnection and introduced urban stressors such as noise, air pollution, and crowding, which pose public health risks (2). In this context, nature contact has become a crucial pathway for health promotion and a core element of nature-based solutions. Observational studies consistently show that greener living environments are linked to lower risks of cardiovascular disease (3), respiratory illness (4), mental health problems (5), and all-cause mortality (6). Experimental research, including controlled trials, has further confirmed the physiological and psychological benefits of nature exposure (7–10). Together, this evidence underscores the significant health value of contact with nature.

Despite the extensive evidence supporting the association between nature exposure and health, the mechanisms underlying this relationship remain not fully understood. A review of theoretical frameworks reveals that whether the focus is on green space (11) or on natural biodiversity (12), physical activity consistently emerges as a key mediating variable linking nature and health. This mediating role becomes particularly salient when considering natural environments within urban settings (71). Building on these theoretical models, many studies have investigated the link between natural environments and physical activity, providing substantial supporting evidence (13–15).

Nevertheless, the specific mechanisms by which green spaces promote physical activity have not been fully elucidated. According to the framework proposed by Markevych et al. (11), green spaces may encourage physical activity primarily by providing safer, less polluted, and thermally comfortable environments. However, in some studies, environmental variables such as air pollution and noise only partially account for the association between green space and physical activity (16). In other words, additional mediating variables need to be considered to further explain the positive link between green or natural environments and physical activity. In this study, we propose that the perceived restorativeness of the environment is a key mediating factor worth investigating.

The concept of restorativeness/restoration—defined as the renewal or recovery of depleted psychological and attentional resources—originates from Stress Reduction Theory and Attention Restoration Theory (17). Stress Reduction Theory posits that environments rich in natural elements can alleviate stress and elicit positive emotional responses (18). Attention Restoration Theory suggests that certain everyday tasks consume directed attention, whereas nature can engage involuntary attention and thus provide opportunities for the restoration of directed attention (19, 20). According to Attention Restoration Theory, a restorative environment should have four core qualities: being away (the ability to mentally and physically escape from routine stressors and obligations), extent/coherence (the richness and coherence of the environment that allows immersive exploration), fascination (soft fascination that captures attention effortlessly without overstimulation), and compatibility (alignment between the environment and one’s purposes and inclinations, encouraging engagement and experience) (20).

Perceived restorativeness is increasingly recognized as a key pathway linking environments with health. Studies show it mediates the effects of biodiversity and naturalness on well-being (21), campus green-space qualities on restoration (22), and campus greenness on student quality of life (23). Together, these findings suggest restorativeness is a common mechanism through which nature enhances mental health, warranting examination of its role in behaviors such as physical activity.

Given the substantial psychological demands of urban life, we argue that perceived restorativeness is a key factor that draws individuals into natural environments for both relaxation and activity. Vegetation, as a quintessential element of nature, plays a vital role in supporting human health (24) and is strongly associated with restorative perceptions (25, 26).

Indeed, several observational studies have suggested that greater vegetation coverage may enhance perceived restorativeness, which in turn could promote physical activity (27, 28). Similar findings are also reported in non-green environments (29). However, given the inherent limitations of observational research—such as residual confounding and difficulties in establishing causal direction—experimental designs with controlled conditions are essential for drawing firmer conclusions.

In this study, we aim to examine the relationship between vegetation, perceived restorativeness, and intention to engage in physical activity through an image-based experimental design with a sample of Chinese university students. Chinese college students often face heavy academic stress and low physical activity levels (30). For this group, restorative environments are especially important to reduce mental fatigue and encourage activity. Studying this population can thus inform campus planning to enhance restoration and promote student physical activity.

Our hypotheses are as follows:

(H1) Higher levels of vegetation will lead to greater perceived environmental restorativeness and a stronger intention to engage in physical activity.

(H2) Perceived environmental restorativeness will mediate the relationship between vegetation levels and physical activity intention.

## 2 Materials and methods

### 2.1 Participants

This study recruited a sample of 633 undergraduate students from a university. Recruitment notices were disseminated through campus communication groups. The primary purpose of the study and the intended use of the data were clearly communicated in the recruitment message. Participation was entirely voluntary and anonymous. All participants were required to provide informed consent prior to completing the questionnaire. The study was reviewed and approved by the Ethics Committee of Southwest University.

### 2.2 Study design and procedure

Given that the Perceived Restorativeness Scale (PRS) assesses subjective perceptions of specific environments, we developed visual stimulus materials for this study.

Drawing on previously validated stimuli (31) and theoretical assumptions from existing literature (32, 33), we created three images depicting varying levels of vegetation—from none to high. To ensure control over confounding variables, we used AI-generated, photorealistic images, following the approach adopted in recent research (34, 35). All images depicted open, boundary-free outdoor spaces. The three images shared a common base of a concrete ground surface, with the amount of visible vegetation progressively increasing and the presence of built/artificial elements decreasing accordingly. Using a concrete base ensured consistency, walkability, and relevance to urban settings.

Participants were then asked to complete an electronic questionnaire on a tablet device. Each participant was randomly assigned one of the three images and instructed to respond based on their imagined experience in that environment.

### 2.3 Variables and measures

#### 2.3.1 Vegetation level

**Figure 1:**
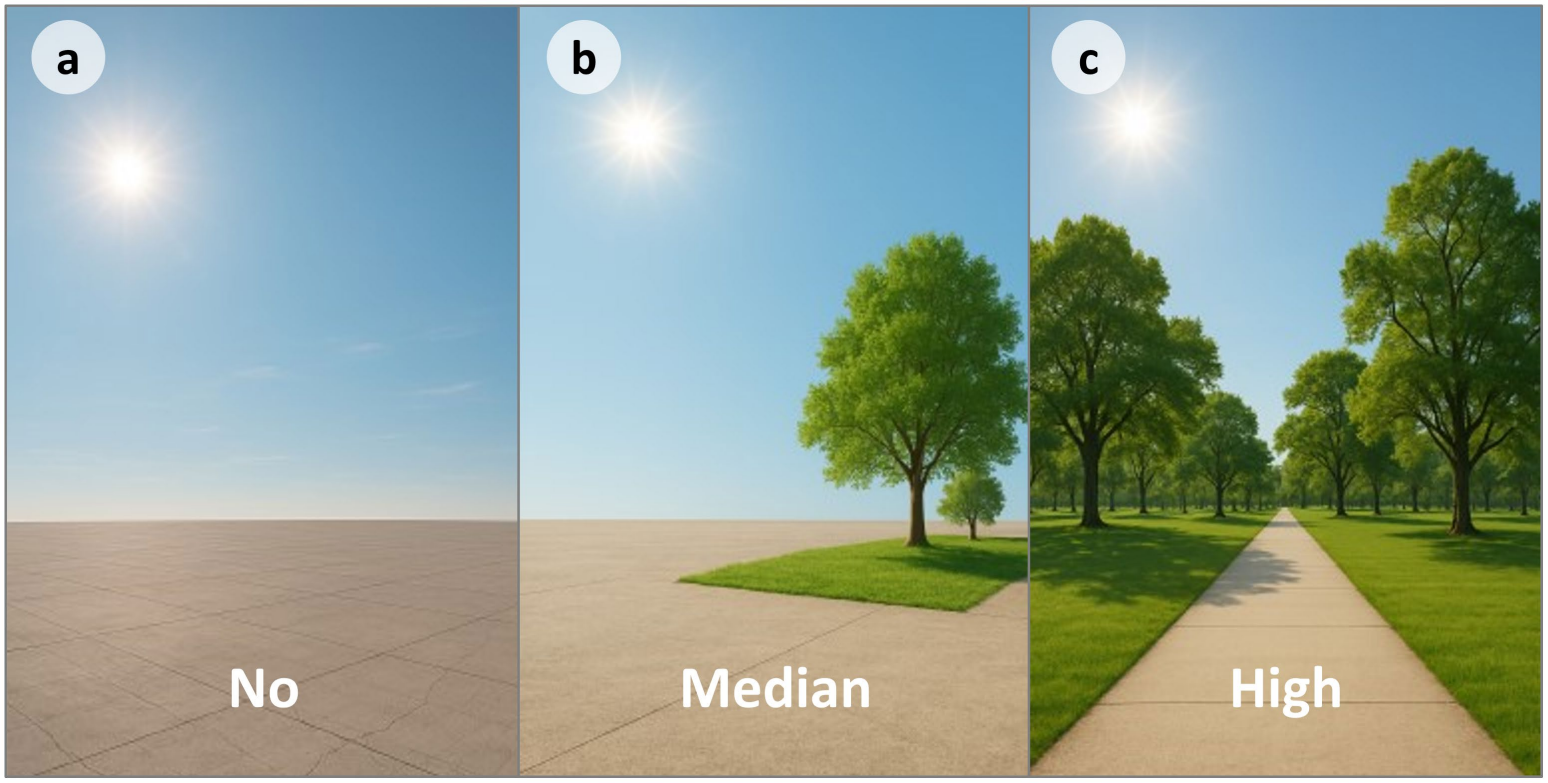
The generated picture stimuli. Panels **(a–c)** represent no, medium, and high vegetation levels, respectively.

According to the image design criteria, the three vegetation conditions were coded as no, medium, and high vegetation levels (Figure 1).

#### 2.3.2 Perceived restorativeness scale (PRS)

We employed the revised 26-item version of the Perceived Restorativeness Scale (PRS) developed by Hartig et al. (36). This version includes a larger number of items, allowing for more flexible item selection in analysis, and features improved wording to enhance readability (e.g., Items 1, 6, and 7). Participants responded using a 7-point Likert scale to indicate the extent to which each statement described their imagined experience in the presented environment (0 = Not at all, 6 = Completely).

The PRS is one of the most widely used instruments for assessing environmental restorativeness. However, it has not been systematically validated within Chinese-language contexts. Therefore, this study first examined the scale’s reliability and validity in our sample. Following best practices in cross-cultural adaptation (37), we employed a translation and back-translation procedure conducted by a team of four bilingual translators. A committee approach was used to reconcile discrepancies and produce the final Chinese version of the scale (see Appendix Table 1).

#### 2.3.3 Intention to engage in physical activity

Participants’ intention to engage in physical activity within the presented environment was assessed using an 11-point Likert scale. A score of 0 indicated no intention, while a score of 10 represented a strong intention to participate in physical activity.

### 2.4 Statistical analyses

#### 2.4.1 Validation of the PRS: reliability and validity assessment

As the PRS has not been systematically validated in a Chinese-language context, we first evaluated its reliability and validity. The specific procedures are outlined below.

##### 2.4.1.1 Construct validity

We employed Confirmatory Factor Analysis (CFA) to assess the structural validity of the measurement tools. The analysis was conducted using Structural Equation Modeling (SEM) with Maximum Likelihood (ML) estimation.

According to the guidelines of the COnsensus-based Standards for the selection of health Measurement INstruments (COSMIN), the sample size should be at least seven times the number of items in the scale when validating structural validity (38, 39). Additionally, based on previous experiences with SEM, a sample-to-parameter ratio of 10:1 is considered ideal (40). Therefore, our sample size meets the analytical requirements.

Concerning structural validity, we referred to the following fit indices and their acceptable thresholds: Standardized Root Mean Square Residual (SRMR) < 0.08; Normed Fit Index (NFI) > 0.90; Tucker-Lewis Index (TLI) > 0.90; Comparative Fit Index (CFI) > 0.90; and Root Mean Square Error of Approximation (RMSEA) < 0.10 (41).

Furthermore, we required the standardized factor loadings to be greater than 0.5, the Average Variance Extracted (AVE) to exceed 0.5, and the Construct Reliability (CR) to be higher than 0.6 to ensure the measurement quality of the model (42, 43).

##### 2.4.1.2 Reliability

We used Cronbach’s alpha to assess internal consistency. The acceptable minimal reliabilities of Cronbach’s alpha is 0.7 (44).

#### 2.4.2 Differences in perceived restorativeness and physical activity intention across vegetation conditions

Given the randomized assignment of environmental scenes, we employed the Kruskal–Wallis *H* test to compare PRS scores and intention to engage in physical activity across the three vegetation conditions. Bonferroni-corrected thresholds were applied to determine the significance of pairwise comparisons.

#### 2.4.3 Mediation analysis

To examine whether perceived restorativeness mediates the relationship between vegetation level and intention to engage in physical activity, we conducted a mediation analysis.

In the mediation analysis, we used total scale scores as continuous variables (28). Consistent with measurement theory, the PRS is a reflective construct, with items reflecting perceived restorativeness; its subscales and total score are reflective composites. Physical activity intention, measured by a single Likert item, is also reflective, while vegetation level was experimentally manipulated and thus not reflective or formative.

The analysis was performed using the Maximum Likelihood (ML) estimator. We applied the bias-corrected bootstrap method (45) with 10,000 replications to generate standard errors and confidence intervals for all paths (46–48), which addresses non-normality in the data. An indirect effect (i.e., a product of coefficients for the constituent links) that significantly deviated from zero was considered evidence of mediation (49, 50).

Since two items were deleted when validating the structural validity of the PRS, we additionally conducted a sensitivity analysis by performing mediation analysis with the total score of the full PRS (including all items) without deletion.

A p-level lower than 0.05 was deemed statistically significant in this study. The statistical analyses were conducted using SPSS version 26.0 (IBM Corp., Armonk, NY, United States) and AMOS version 26.0 (IBM Corp.).

## 3 Results

### 3.1 Participant characteristics

In the final sample, male participants slightly outnumbered female participants, comprising 59.4% of the total (Appendix Table 2). More than half of the students were in their second year of undergraduate study. The majority reported a household monthly income within the range of 0–10,000 RMB (approximately 0–1,400 USD as of June 2025). Due to the randomized image assignment design, the number of participants exposed to each of the three vegetation-level scenarios was approximately equal.

### 3.2 Reliability and validity of the PRS

When all items were loaded onto the theoretical structure, the model demonstrated suboptimal fit indices (SRMR = 0.100; NFI = 0.874; TLI = 0.875; CFI = 0.887; RMSEA = 0.108). We identified that Items 10 and 13 had factor loadings below 0.50 (see Appendix Figure 1), and therefore, these items were removed from further analysis. After their removal, model fit improved and met acceptable thresholds (SRMR = 0.724; NFI = 0.910; TLI = 0.913; CFI = 0.922; RMSEA = 0.095). In addition, both Average Variance Extracted (AVE) and Composite Reliability (CR) values met the recommended criteria (see Appendix Figure 2).

It is worth noting that the inter-factor correlation between “being away” and “fascination” were relatively high, approaching the liberal threshold of concern (*r* = 0.9) (Appendix Figure 2), as suggested by others (51, 52). We further tested discriminant validity. By the Fornell–Larcker criterion (53), AVE values were lower than squared inter-factor correlations, indicating limited discriminant validity. Item-level checks showed no major cross-loadings; only two items slightly exceeded 0.30 on non-theorized factors, well below the 0.40 threshold (54–56) and much lower than their intended loadings (Appendix Figure 3).

### 3.3 Differences in perceived restorativeness and intention to engage in physical activity across vegetation scenarios

**Figure 2:**
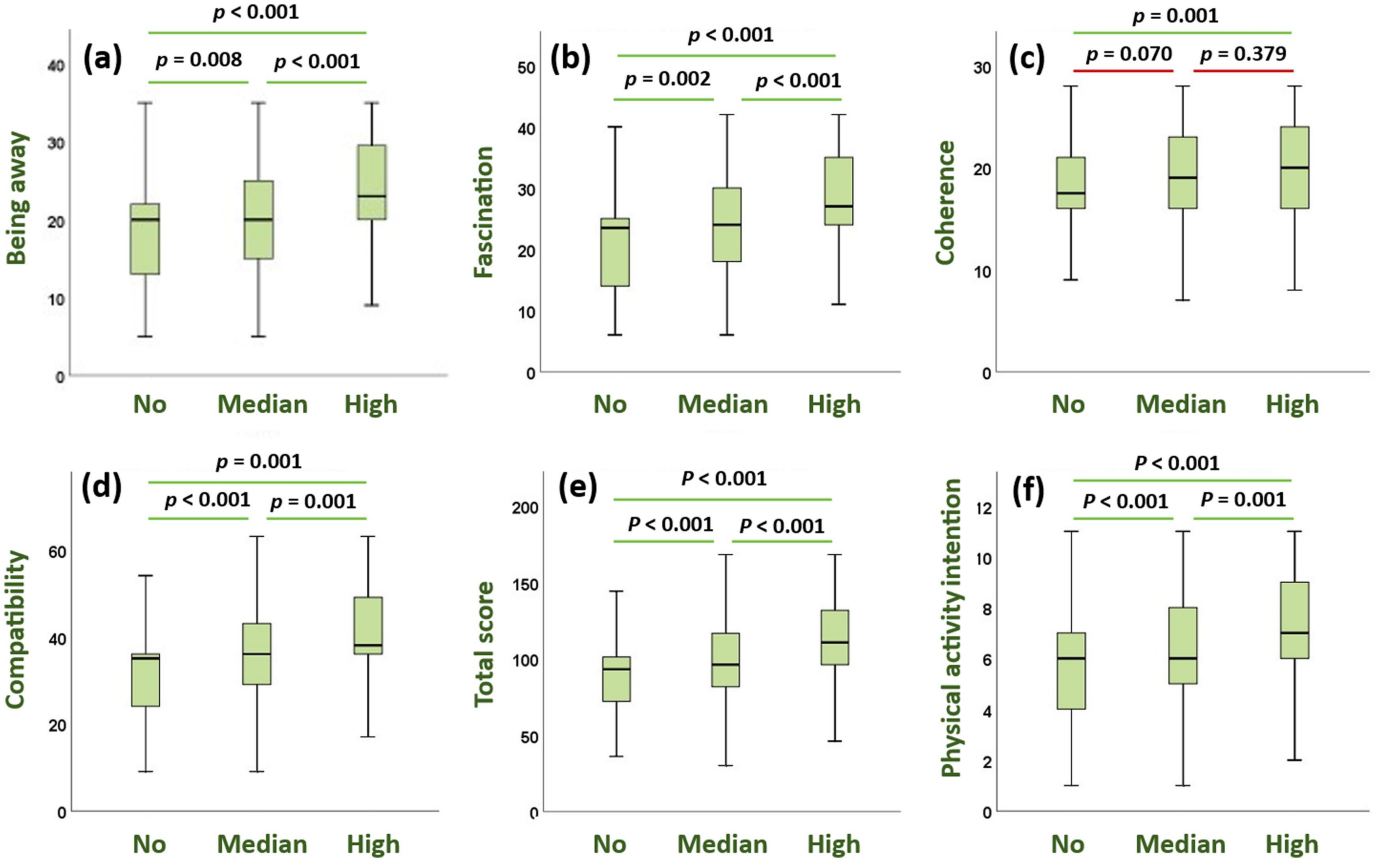
Differences in restorativeness and intention to engage in physical activity scores across vegetation levels. Boxes represent the interquartile range (IQR) with the horizontal line indicating the median, whiskers representing 1.5 × IQR; *p*-values above the boxes denote significant differences between groups based on Kruskal–Wallis tests with Bonferroni-adjusted pairwise comparisons. The **(a-f)** represent the outcome variables are loaded as being away, fascination, coherence, compatibility, the total score of PRS, and physical activity intention.

Kruskal–Wallis tests showed significant differences across vegetation conditions for Being Away (*H* = 61.29, *p* < 0.001, *η*^2^ = 0.094), Fascination (*H* = 68.86, *p* < 0.001, *η*^2^ = 0.106), Compatibility (*H* = 71.60, *p* < 0.001, *η*^2^ = 0.110), PRS total (*H* = 84.08, *p* < 0.001, *η*^2^ = 0.130), and physical activity intention (*H* = 54.40, *p* < 0.001, *η*^2^ = 0.081). Coherence showed only a small effect (*H* = 14.59, *p* = 0.001, *η*^2^ = 0.020), with significant differences limited to the comparison between the no-vegetation and high-vegetation conditions (Figure 2).

### 3.4 Mediation analysis

**Figure 3:**
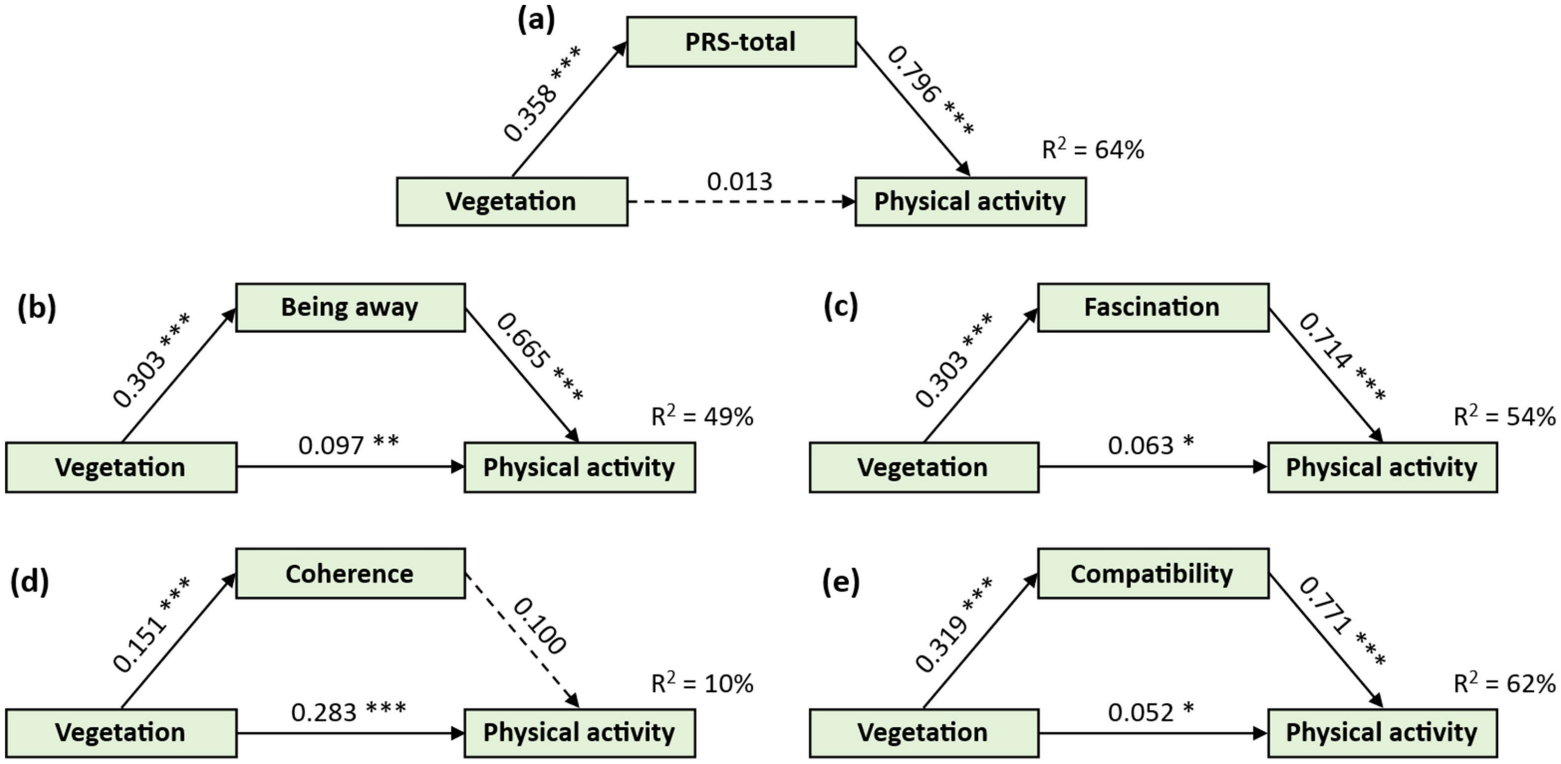
Mediation model of perceived restorativeness between vegetation level and intention to engage in physical activity. *p < 0.05, **p < 0.01, ***p < 0.001; Physical activity, intention to engage in physical activity; PRS-total, the total score of the PRS scale; Dashed lines represent paths with *p* > 0.05. Numbers in the figure indicate standardized regression coefficients. The **(a-f)** represent the outcome variables are loaded as being away, fascination, coherence, compatibility, and the total score of PRS.

Mediation analyses showed significant indirect effects for Being Away (*β* = 0.201), Fascination (*β* = 0.235), and Compatibility (*β* = 0.246; all *p* < 0.001), explaining 67–83% of the total effect (Table 1). Coherence had only a marginal effect (*β* = 0.015, *p* = 0.044; 5.03%). Using the PRS total score, the indirect effect absorbed nearly all of the association (*β* = 0.285, *p* < 0.001; 95.64%), while the direct path was non-significant (*β* = 0.013, *p* = 0.600) (as indicated by the pathways between variables in Figure 3), indicating full mediation.

**Table 1:** Total and indirect (mediation) effects in the mediation models.

Mediator	Effect	*β*	95% CI		*p*	Mediation rate
			Lower	Upper		
Being away	Total	0.298	0.224	0.367	<0.001	–
	Indirect	0.201	0.155	0.248	<0.001	67.45%
Fascination	Total	0.298	0.224	0.367	<0.001	–
	Indirect	0.235	0.184	0.286	<0.001	78.86%
Coherence	Total	0.298	0.224	0.367	<0.001	–
	Indirect	0.015	0.000	0.039	0.044	5.03%
Compatibility	Total	0.298	0.224	0.367	<0.001	–
	Indirect	0.246	0.189	0.302	<0.001	82.55%
PRS-total	Total	0.298	0.224	0.367	<0.001	–
	Indirect	0.285	0.231	0.338	<0.001	95.64%

β = standardized regression coefficient.

### 3.5 Sensitivity analysis

Sensitivity analysis using all PRS items yielded nearly identical results (Appendix Figure 4): total effect *β* = 0.298 (95% CI = 0.224–0.367, *p* < 0.001), indirect effect *β* = 0.288 (95% CI = 0.235–0.340, *p* < 0.001), with mediation efficiency of 96.64%.

## 4 Discussion

### 4.1 General discussion

In this study, we validated the reliability and validity of the Chinese version of the Perceived Restorativeness Scale (PRS) in a sample of Chinese university students. Using this tool, we examined the pathway linking vegetation levels to intention to engage in physical activity through perceived restorativeness. To minimize confounding factors inherent in observational studies, we employed an image-based experimental design to collect data. Our main findings indicate that higher vegetation levels are associated with greater perceived restorativeness and increased intention to engage in physical activity. Furthermore, perceived restorativeness plays a significant mediating role in the relationship between vegetation level and physical activity intention. Notably, when using the total PRS score as the mediator, we observed a full mediation effect, as evidenced by the near-zero direct effect after including the mediator (57). Although this does not completely exclude the possibility of other mediating factors (58), it does provide strong evidence supporting the critical role of perceived restorativeness in linking vegetation levels and physical activity intention.

It is noteworthy that when Coherence was modeled as the mediator, its effect was weak. Differences appeared only between no- and high-vegetation conditions, implying that order and legibility emerge mainly with abundant vegetation. We assume that coherence may also be less relevant for motivating activity than fascination or compatibility. Methodological factors—such as simple AI-generated images and high inter-factor correlations—may have further reduced its role. Future studies with richer, multi-sensory stimuli should test whether this reflects theory or method.

This study measured physical activity intention rather than behavior. Although intention is a key predictor, the intention–behavior gap means strong intentions may not translate into action (59, 60). Thus, our findings reflect motivation rather than behavior. Future work should include objective or self-reported measures (e.g., accelerometry, EMA, follow-ups) to test whether restorative environments increase actual activity.

In summary, these findings largely support our Hypotheses 1 and 2 and offer a theoretical foundation for understanding how green spaces promote or stimulate physical activity.

### 4.2 Application of the PRS in the Chinese context

Although the validation of the PRS was not the primary focus of this study, it represents a necessary step to enhance internal validity and demonstrate the credibility of our evidence. The PRS is one of the earliest and most widely used instruments for measuring environmental restorativeness (61); however, its validation within Chinese settings remains very limited. Many studies have directly employed Chinese translations of the PRS without further examination of its reliability and validity (62–64). Through a literature review, we identified only one earlier Chinese study that used exploratory factor analysis to preliminarily examine an earlier version of the PRS with fewer items (65). The revised version employed in our study, however, had never been translated or psychometrically tested previously.

In this study, we observed satisfactory structural validity; nevertheless, the high inter-factor correlations suggest that the original theoretical structure might face challenges. Despite this, drawing on the concept of known-groups validity—which posits that measurement outcomes should reflect differences between theoretically distinct groups (66)—the observed differences in PRS subscale and total scores across different vegetation scenarios further support the scale’s measurement validity.

In conclusion, further validation work is warranted, such as conducting exploratory and confirmatory factor analyses in larger and more representative populations.

### 4.3 The mediating role of environmental restorativeness between vegetation level and physical activity intention

To date, numerous studies have examined the association between vegetation levels and physical activity (13, 16, 62). However, these studies often employed observational designs, which cannot rule out confounding factors or reverse causality—for example, individuals with more resources for physical activity might be more likely to live in greener neighborhoods. Moreover, as Markevych et al. (11) pointed out, previous research rarely distinguished the specific locations where physical activity occurred, potentially recording some activities unrelated to green spaces, thereby distorting findings. In contrast, this study used a design similar to a randomized controlled trial and specifically assessed participants’ intentions to engage in physical activity in defined scenes. These findings reinforce and refine the understanding that green spaces can promote physical activity. It should be noted, however, that since our study is still based on surveys rather than actual behavioral experiments, we measured physical activity intention rather than objectively measured physical activity levels. Therefore, these findings cannot fully explain the observed promotion effect of green spaces on physical activity.

Nevertheless, our findings provide an important mechanism to explain the relationship between green space and physical activity, especially as we found a full mediating effect. Prior to this study, numerous investigations confirmed a positive association between vegetation and perceived environmental restorativeness (67, 68). However, only a few studies attempted to further link environmental restorativeness with physical activity (27, 28). Our study further validates this indirect pathway and emphasizes that residents’ pursuit of psychological restoration opportunities is a key reason for their engagement in green space activities.

It should be noted that data were collected near final exams, when stress and fatigue are high. This may have heightened sensitivity to restorative environments and inflated restorativeness and intention scores, potentially overestimating the mediation effect. Future studies under more neutral conditions are needed to confirm robustness.

### 4.4 Research contributions

This study has two main contributions:

(1) Testing and revising the Perceived Restorativeness Scale (PRS) in the Chinese context.
(2) Identifying a strong mediating effect of environmental restorativeness between vegetation level and physical activity intention, providing a mechanistic insight into the “green space—physical activity” association.

Beyond the university context, our findings suggest that urban planners, public health practitioners, and campus designers could leverage vegetation to create environments that simultaneously foster restoration and physical activity. Investing in greener spaces is not merely aesthetic but represents an evidence-based strategy to support healthier, more active communities.

### 4.5 Limitations

A limitation concerns the ecological validity of our experimental design. Although the use of images as stimuli is common in this type of research, it may reduce overall perceived restorativeness judgments and neglect other sensory modalities. Prior work has shown that image-based methods can underestimate restorativeness compared to real-world environments (69). Moreover, Grahn has emphasized that sensory inputs beyond vision—such as sound—are essential for stress restoration (70). As a result, our findings should be interpreted as reflecting responses to visual aspects of vegetation rather than the full spectrum of restorative experiences. Future research employing field experiments or immersive technologies (e.g., virtual or augmented reality) could enhance ecological validity and more accurately inform health promotion and urban design practices.

Second, to control environmental variables, we used simulated rather than real-scene photographs, which may affect the authenticity of the experience. Third, we used a convenience sample from only one university, limiting representativeness. Future research should consider broader populations. Finally, some deficiencies exist in the reliability and validity of the scales used, which restrict the internal validity of the study. Another limitation relates to sample composition. Male students accounted for nearly 60% of participants, creating a gender imbalance that may have influenced PRS responses and physical activity intentions.

## 5 Conclusion

This study validated the reliability and validity of the Chinese version of the Perceived Restorativeness Scale (PRS) in a sample of Chinese university students and further revealed the mediating role of environmental restorativeness between vegetation levels and intention to engage in physical activity. By using simulated image-based experiments, we effectively controlled for potential confounding variables and found that higher levels of vegetation significantly enhanced both perceived restorativeness and intention to be physically active. More importantly, the total PRS score showed a full mediating effect between vegetation level and physical activity intention, suggesting that individuals may be more intending to engage in physical activity in highly vegetated environments partly because they perceive greater psychological restoration in such settings. These findings not only support the application of environmental restorativeness theory in the context of physical activity research but also provide empirical evidence for the psychological mechanisms underlying the “green space–health behavior” link. Future studies should consider extending the sample population and adopting more ecologically valid research designs (e.g., field experiments or behavioral tracking) to further test the applicability of the proposed mechanism across broader contexts.

## Data Availability

The raw data supporting the conclusions of this article will be made available by the authors, without undue reservation.
