# Supplementary material for: The mediating role of environmental restorativeness between vegetation levels and physical activity intention: a photo-based experimental study
Source: Front Public Health. 2025 Oct 3;13:1653065. doi: 10.3389/fpubh.2025.1653065 (PMC12531268; doi:10.3389/fpubh.2025.1653065)
Supplement: Supplementary file 1 [file Data_Sheet_1.docx]

Supplementary Material

**Table 1.** The translated scale

| **Original items** | **Final translation** |
| --- | --- |
| 1.Being here is an escape experience. * | 1.在这里有种远离世俗的体验。 |
| 2.Spending time here gives me a break from my day-to-day routine. † | 2.待在这里能让我从日常事务中喘口气。 |
| 3.It is a place to get away from it all. | 3.这里是个能让我远离一切的地方。 |
| 4.Being here helps me to relax my focus on getting things done. | 4.在这里，我不用总是想着要完成任务，可以放松下来。 |
| 5.Coming here helps me to get relief from unwanted demands on my attention. | 5.来这里可以帮助我摆脱那些让我分心的事情。 |
| 6.This place has fascinating qualities. * | 6.这个地方有令人着迷的特点。 |
| 7.My attention is drawn to many interesting things. * | 7.在这里，有很多有趣的东西吸引我的注意力。 |
| 8.I want to get to know this place better. † | 8.我想更好地了解这个地方。 |
| 9.There is much to explore and discover here. * | 9.这里有很多值得探索和发现的东西。 |
| 10.I want to spend more time looking at the surroundings. † | 10.我想多花点时间观看周围的环境。 |
| 11.This place is boring. (-) | 11.这个地方很无聊。 |
| 12.The setting is fascinating. | 12.这里的环境很吸引人。 |
| 13.There is nothing worth looking at here. (-) | 13.这里没什么值得看的。 |
| 14.There is too much going on. (-) * | 14.这里太多事情在发生，太乱了。 |
| 15.It is a confusing place. (-) * | 15.这个地方让我感到迷惑。 |
| 16.There is a great deal of distraction. (-) * | 16.这里有太多东西会让我分心。 |
| 17.It is chaotic here. (-) * | 17.这里很混乱。 |
| 18.Being here suits my personality. * | 18.待在这里是符合我的性格的。 |
| 19.I can do things I like here. * | 19.在这里我可以做自己喜欢的事情。 |
| 20.I have a sense that I belong here. * | 20.我有种属于这里的感觉。 |
| 21.I can find ways to enjoy myself here. † | 21.在这里，我能找到让自己快乐的方法。 |
| 22.I have a sense of oneness with this setting. * | 22.我觉得自己和这的环境很契合，像是一体的感觉。 |
| 23.There are landmarks to help me get around. ‡ | 23.这里有一些明显的地标性事物，能方便我四处走动。 |
| 24.I could easily form a mental map of this place. ‡ | 24.我能很容易在心里想象出这个地方的地图。 |
| 25.It is easy to find my way around here. ‡ | 25.这里很好找路，不容易迷路。 |
| 26.It is easy to see how things are organized. ‡ | 26.我很容易就可以看出这里是如何布局和设置的。 |

Notes: (-) an item for which the value must be reversed in coding; (*) the item is from the

earlier PRS and is in its original form; (†) the item is from the earlier PRS but is in a revised

form; (‡) the item was designed to represent legibility

**Table 2.** Participants’ characteristics and measures of vegetation level and intention to engage in physical activity

| Variable | Category/Unit | N (%) | Mean (SD) |
| --- | --- | --- | --- |
| Sex | Male | 376 (59.4%) | - |
|  | Female | 257 (40.6%) | - |
| Grade | First | 156 (24.6%) | - |
|  | Second | 408 (64.5%) | - |
|  | Third | 44 (7.0%) | - |
|  | Fourth | 25 (4.0%) | - |
| Family income | 0-5000 RMB/Month | 300 (47.4%) | - |
|  | 5001-10000 | 218 (34.4%) | - |
|  | 10001-15000 | 68 (10.7%) | - |
|  | 15001-20000 | 22 (3.5%) | - |
|  | 20001-25000 | 4 (0.6%) | - |
|  | 25001-30000 | 2 (0.3%) | - |
|  | > 30000 RMB/Month | 19 (3.0%) | - |
| Vegetation level | No | 206 (32.5%) | - |
|  | Medium | 211 (33.3%) | - |
|  | High | 216 (34.1%) | - |
| Age | Years | - | 20.22 (1.40) |
| Intention to engage in physical activity | Score | - | 6.50 (2.64) |


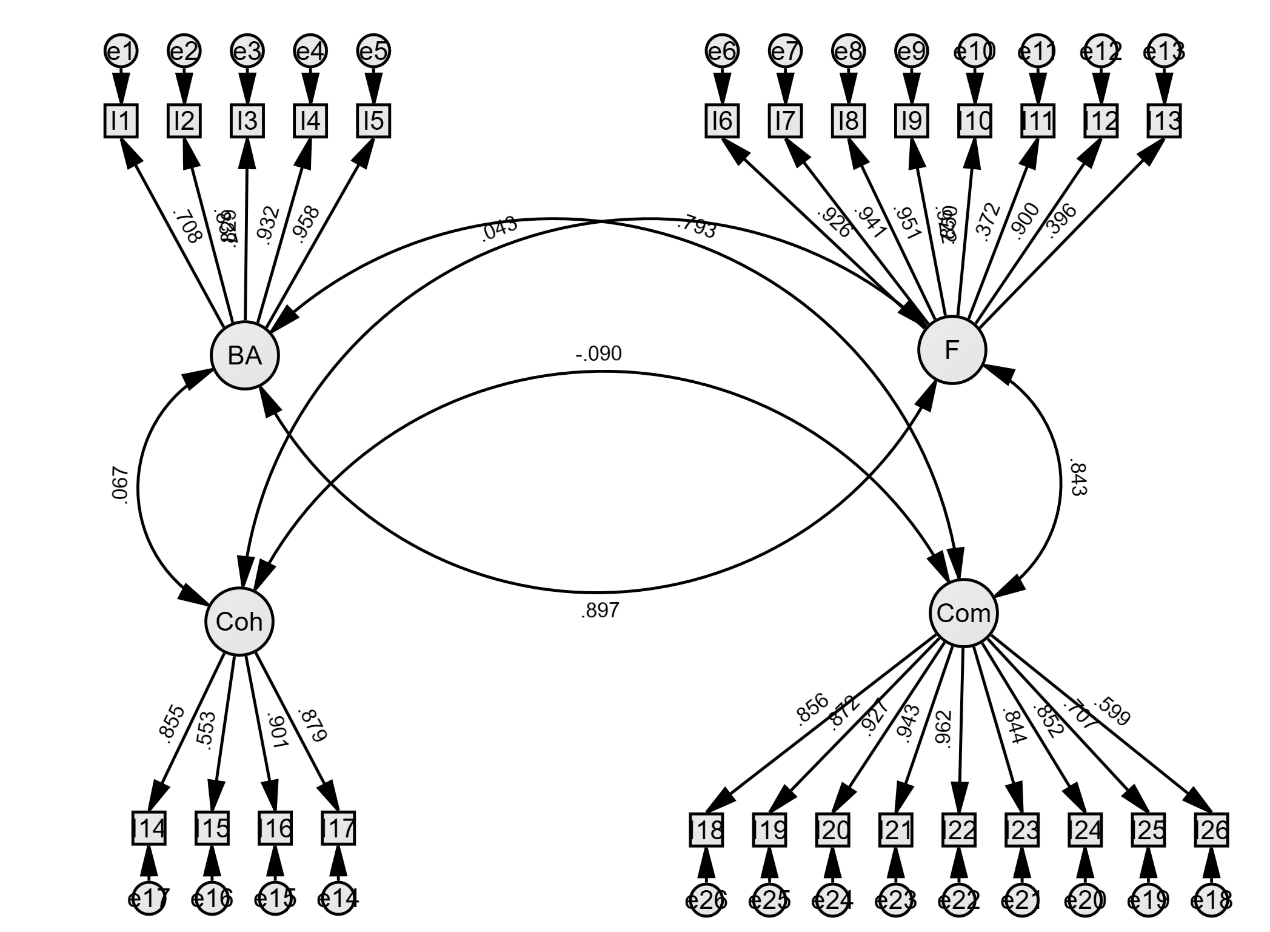


**Figure 1.** Initial model with all 26 factors

Note: BA, being away; F, fascination; Coh, coherence; Com, compatibility


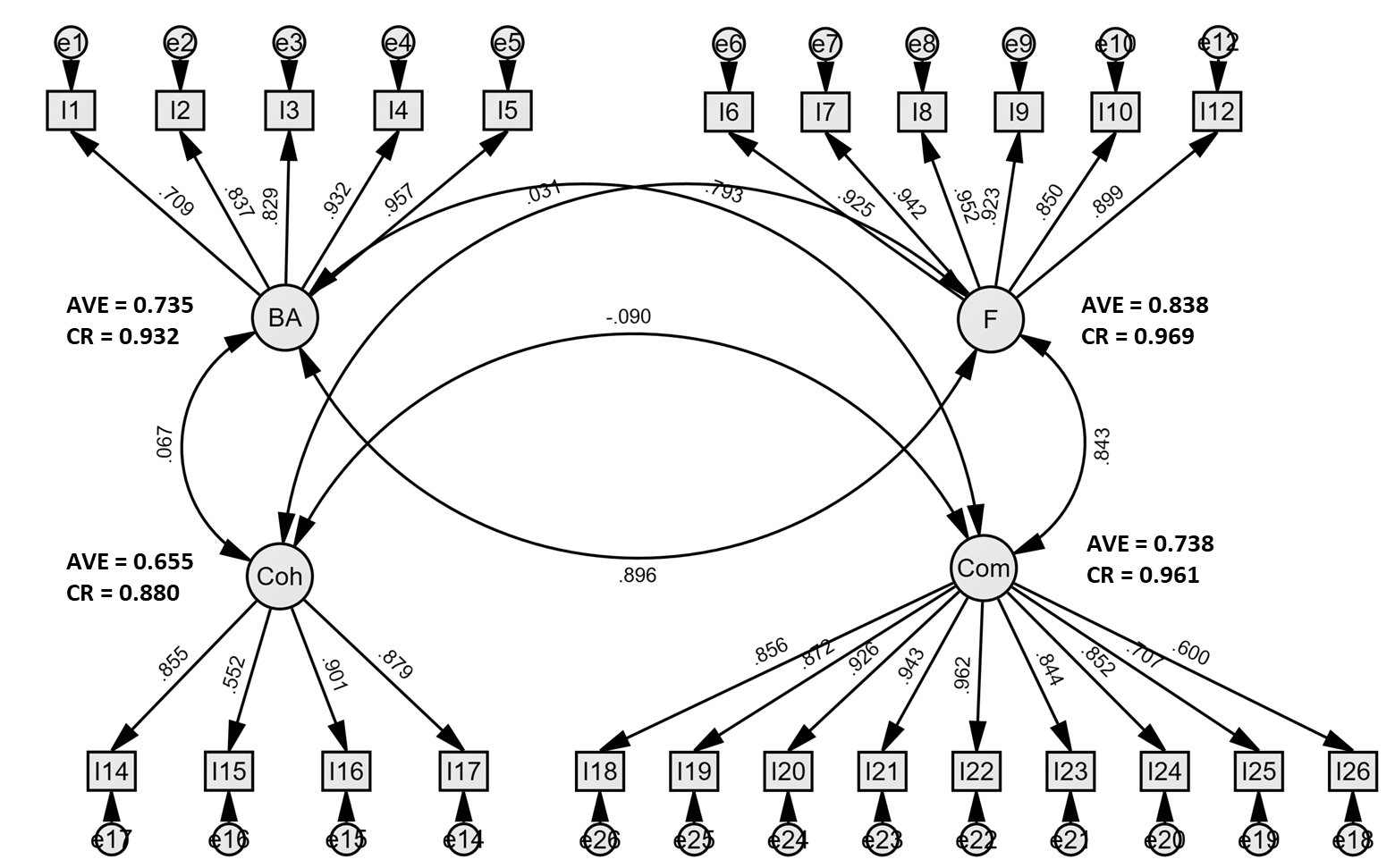


**Figure 2.** The final CFA model after modifications

Note: BA, being away; F, fascination; Coh, coherence; Com, compatibility


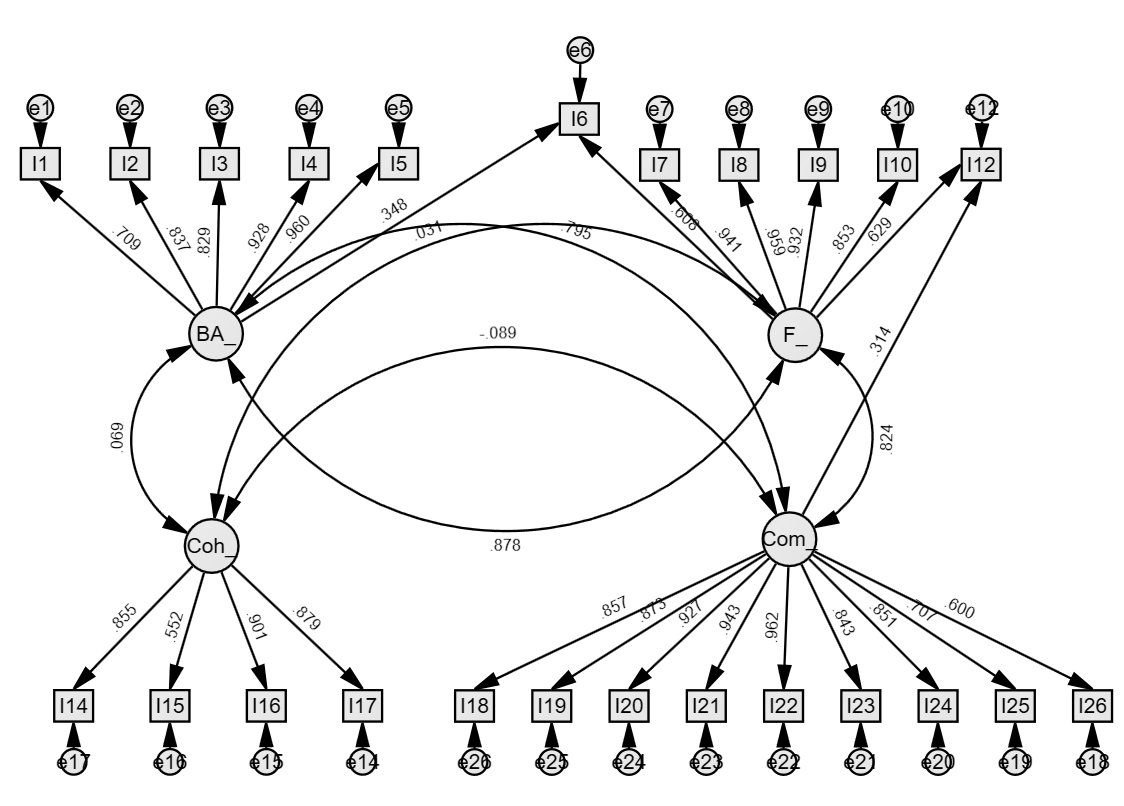


**Figure 3.** Cross-loading values of items

Note: BA, being away; F, fascination; Coh, coherence; Com, compatibility


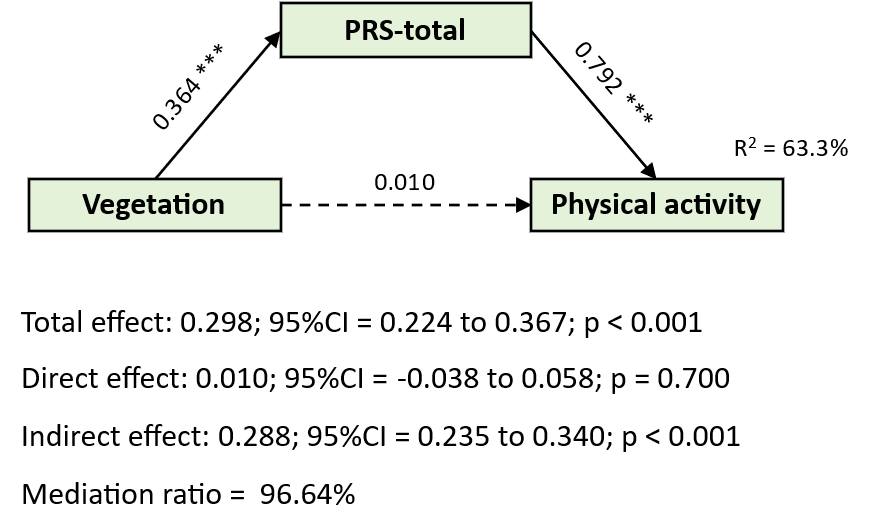


**Figure 4.** Sensitivity analysis with all PRS items

Note: *, p < 0.05; **, p < 0.01; ***, p < 0.001; Physical activity, intention to engage in physical activity; PRS-total, the total score of the PRS scale; Dashed lines represent paths with p > 0.05. Numbers in the figure indicate standardized regression coefficients.
